# Climate change, urbanization and disease: summer in the city…

**DOI:** 10.1093/trstmh/tru194

**Published:** 2014-12-08

**Authors:** Robert C. Reiner, David L. Smith, Peter W. Gething

**Affiliations:** a Fogarty International Center, National Institutes of Health, Bethesda, MD 20892, USA; b Department of Epidemiology and Biostatistics, Indiana University School of Public Health, Bloomington, IN 47401, USA; c Spatial Ecology and Epidemiology Group, Tinbergen Building, Department of Zoology, University of Oxford, South Parks Road, Oxford, UK

**Keywords:** Climate change, Composition of disease, Seasonality, Tropical climates, Urbanization

## Abstract

Climate change and urbanization can alter the burden of human diseases. The tropics, a region that includes the poorest populations and highest disease burdens, are expected to get slightly hotter and substantially more urban. Studies have projected changing burdens under different climate or urbanization scenarios, but it remains unclear what will happen if both happen at once. Interactions could amplify disease burdens, improve health overall, or shift burdens around. Social planners need better data on contemporary seasonal disease incidence patterns across the spectrum of climate, urbanicity and socio-economic status. How climate change, urbanization and health interact must be understood to adequately plan for the future.

## Background

Cities are growing and many rural or peri-urban areas are becoming urban. By 2050, 60% more people will live in urban areas than today.^1^ In tropical climates within Asia and Africa, the proportion of the urban population is increasing by 1.5 and 1.1% per annum, respectively.^1^ Over the same time period, depending on the season and exact location within the tropics, the mean surface temperature is expected to rise by between 1 and 3°C.^2^ Urbanization and climate change could each alter the composition, intensity and seasonality of pathogen transmission and disease incidence across the tropics, but since both changes will occur at once, the drivers of these disease systems will interact in complex ways to determine what diseases are present and the corresponding seasonality of infection, disease and death.

The tropics include much of the world's rural population as well as many of the fastest growing urban centers.^1^ In addition to chronic diseases that afflict more industrialized areas, these areas are disproportionately burdened by infectious diseases.^3^ While climate change and urbanization could act synergistically to increase the burden of current endemic diseases within densely populated urban cores, they could also result in complex (and possibly antagonistic) outcomes. These effects are most unpredictable in rural and peri-urban locations undergoing the transition into more developed, prosperous cities.

The timing and magnitude of seasonal patterns in all-cause mortality is, of course, driven at any given location by the particular local mix of causes of death, and their individual seasonal characteristics. This further complicates predictions of the impact of changing climate and urbanization. Even if the likely effects on major causes of death could be predicted, their co-distributions, interactions and overall impact on mortality, and seasonality of mortality, would vary geographically. This is made yet more complex by the fact that both urbanization and climate change may alter ranges or occurrence rates of both infectious and chronic diseases.^3,4^

## Urban cores and megacities

Many of the current large urban areas in the tropics are undergoing relatively unchecked growth, with population increases well outpacing improvements in infrastructure. Megacities, such as Dhaka or Jakarta as well as megacities-in-waiting, such as Kinshasa, are overwhelmed with a seemingly unending array of public-health threats.^5^ Pollution, a major driver of respiratory-related diseases, will almost surely become a larger problem with further urbanization and increasing temperature. Increases in population density combined with warmer climates will increase the number of individuals without reliable access to freshwater^6^ and will exacerbate projected increases in heat-related deaths.^4^ Poor sanitary conditions driven by ineffective infrastructure and potential increases in flooding will combine within megacities to drive the incidence of diarrheal diseases like cholera and rotavirus.^7^ With global climate change projected to increase the frequency of extreme weather events,^2^ diarrheal diseases, the leading killer of children under the age of 5 years, will maintain their endemic status and strongly drive patterns of mortality.

Chronic diseases such as respiratory and cardiovascular diseases are constant threats within urban cores that are strongly influenced by increases in temperature.^4^ Their contribution to disease-induced deaths will likely increase during the hotter months of the year. The seasonality of many infectious diseases are also strongly linked to climatic drivers, but no single climatic driver affects all infectious diseases in the same way. Shigella bacteria favor moderate temperatures, so shigella-related deaths would increase in places that are now colder.^4^ Conversely, warmer temperatures tend to favor both mosquitoes and the pathogens they transmit, but only up to a point. Though the burden of dengue is increasing, it remains unclear whether this is primarily the result of urbanization or climate change. Malaria, on the other hand, tends to decline with urbanization^8^ although urban malaria can be a significant health risk in some settings.^3^ Co-infection and comorbidity, both of which are a major threat in urban cores, will alter the overall disease-related death rates in ways that are difficult to predict and likely not universal across all large cities within the tropics.

## Rural villages and peri-urban cities

Climate change and population growth may not always impact the timing and composition of disease within villages and small cities in the same way. As a village develops into a city there are typically increases in access to healthcare, socio-economic status and disease control efforts. The speed at which a region urbanizes (or rather the length of the transition from rural to developed) can be associated with a potentially decade-long transient period where disease incidence actually increases.^9^ While chronic diseases associated with pollution may not be as relevant in small cities, poor access to adequate drinking water can still pose a large health risk.^6^

Several studies have found that development of a region has resulted in a decrease in the intra-annual variation in disease occurrence. Frequently this is due to an improvement in healthcare access,^4^ but it can also be due to a stabilization of disease reservoirs as a response to human-induced land change or irrigation.^9^ Although individual diseases are still influenced by climatic drivers such as rainfall and temperature, an increase in access to healthcare appears to mute the seasonal signal of disease and disease-related death. While improved access to healthcare is of an obvious benefit to the public, it unfortunately masks the proximal mechanisms driving temporal variation in incidence. As such, to adequately account for the role these drivers and the changing landscape plays on the seasonality of disease-related deaths, even more data is required.

## Conclusions

Understanding contemporary seasonal patterns of all disease-induced deaths is vital for adequate public health responses, but so are investigations of individual causes. Unfortunately, many cities' clinics and hospitals are routinely overwhelmed by infectious disease outbreaks (e.g., Dhaka during cholera season), and accurately predicting when resources should be increased (as well as possibly when resources are needed elsewhere) can greatly reduce the number of disease cases that result in death. Additionally, from the standpoint of control, accurately identifying times of the year where the effect of control efforts will be maximizing can greatly reduce mortality.^10^ Both climate change and urbanization will greatly alter an already poorly understood landscape. As population densities increase, planning could improve the responses of overstretched health systems, but this places greater emphasis on understanding the world today. At present, the necessary data and models with which to provide this information is lacking.
